# Patient-reported outcomes feedback report for knee arthroplasty patients should present selective information in a simple design - findings of a qualitative study

**DOI:** 10.1186/s41687-020-0173-7

**Published:** 2020-01-21

**Authors:** Kathrin I. Fischer, Diarmuid De Faoite, Matthias Rose

**Affiliations:** 1 Charité – Universitätsmedizin Berlin, corporate member of Freie Universität Berlin, Humboldt-Universität zu Berlin, and Berlin Institute of Health, Center of Internal Medicine and Dermatology, Department of Psychosomatic Medicine, Berlin, Germany; 2 Smith & Nephew, Clinical Scientific and Medical Affairs, Global Clinical Strategy, Baar, Switzerland; 3 Department of Quantitative Health Sciences, University of Massachusetts Medical School, Worcester, MA USA

**Keywords:** Quality of Life, Feedback Report, mHealth, Patient-reported Outcome Measures, Knee Arthroplasty

## Abstract

**Background:**

Technical innovation to assess patient-reported outcomes (PROs) facilitates their implementation in clinical practice. In particular, mobile applications (apps) allow PROs to be assessed outside of the clinical setting. A patient’s health status can be remotely monitored and evaluated after discharge, and their recovery process tracked. This is of particular interest for patients after knee arthroplasty, as the recovery phase after surgery usually takes place in an outpatient setting and requires a high level of patient engagement. Providing results of PRO assessments to patients in the form of a feedback report could increase patient engagement and may improve communication between health care professionals and patients. The aim of the study is to develop a PRO feedback report for mobile devices that is comprehensible and provides valuable information for patients after knee arthroplasty.

**Results:**

In an iterative development process, our expert group developed two preliminary feedback reports (a text-based version and a graphical display) based on previous research results and practical experience. In a second step, we discussed these reports with orthopedic patients (*n* = 8) in terms of comprehensibility and value using semi-structured interviews and cognitive debriefing methods. Participants assessed the reports as informative, but had some difficulties in fully comprehending all of the information provided. Based on the feedback from patients, we modified both versions and reduced complexity to increase comprehensibility.

**Conclusions:**

A PRO feedback report for patients for mobile app use has to take account of the heterogeneous user group, particularly demographics such as age and experience with mobile devices. Information should be presented in a simple way to be comprehensible and of value to patients. Technological advancements allow a simple default report to be set, something which enables patients interested in additional information to make customizations.

## Background

Patient-reported outcomes (PROs) play an increasingly important role in health care [1]. In clinical practice, PROs are used to assess the health status and wellbeing of patients [2, 3]. In addition, PROs are used as screening and monitoring tools to support health care professionals in providing patient-centered care and facilitate communication between patients and health care professionals [1, 2, 4].

Electronic assessment of PROs is becoming established in research and clinical practice, not in the least because scores can be automatically calculated and provided in real-time to health care professionals [5, 6]. Moreover, mHealth technologies, referring to health care delivery via mobile devices [7], enable PRO assessments at home [8–11]. The increasing usage of smartphones in all age groups [12] and the high potential seen in mHealth solutions also for an older generation [13], support the new direction of PRO data collection using mobile devices. In this way, health care professionals are able to monitor patients, evaluate their health status and communicate with patients, outside of the clinic [10].

The provision of PRO data to patients, in the form of an automatically generated PRO feedback report, which can be directly accessed by patients after completion, is not common practice. Health care professionals frequently provide information on PRO results during the consultation [6, 14, 15]. In an outpatient setting, patients thereby have delayed access to information on their PRO results, if PROs are collected off-site. However, timely access to PRO scores enables patients to reflect on their progress and promotes active management of their health situation [3].

In orthopedics, the advancement of surgical techniques, as well as cost pressures in clinics, has led to a decrease in the length of hospital stays [16, 17]. The majority of the recovery time after knee replacement takes place in an outpatient setting, which requires a substantial degree of personal responsibility and active disease management by the patient [18]. One way of supporting orthopedic patients to actively manage their recovery phase after surgery is through early health education and training as well as access to health information such as PRO results by providing mHealth solutions [19–22].

A newly developed education app for knee replacement patients was introduced to support them during their treatment. The app includes information about the post-surgery recovery phase, such as advice on physical activity after surgery, medication and healthy diet. In addition, the PROMIS® measures of Physical Function and Pain Interference are included in the app. Patients are invited to answer these measures at predefined measurement time points. Currently, the PRO scores are only shown in the health care professionals’ dashboard. This information is used to track the recovery phase of individual patients and patient groups. Integration of a PRO feedback report into the app would allow patients to access and monitor their PRO results as well. This information could facilitate patient engagement, improve communication with health care professionals as the PRO feedback report could support patients to address health issues and concerns, based on PRO scores [23].

An automatically generated PRO feedback report for patients using the app is possible, and enables patients to access their PRO results immediately via smartphone. This raises the questions of what kind of information is of value to patients and how a report should look like in order to be easy to understand and evaluate.

## Methods

### Aim

The aim of the study is to develop a PRO feedback report for mobile devices, which can be integrated into the newly developed patient education app for patients after knee arthroplasty. This PRO feedback report should be comprehensible and provide valuable information for adult patients after knee arthroplasty, using the PROMIS® health domains of Physical Function and Pain Interference.

### Development process of a PRO feedback report for patients

In a first step, we conducted a literature review to examine examples and results of PRO feedback reports that provides the basis for the subsequent development steps. The focus of the literature review was to identify publications describing any kind of PRO feedback report for health care professionals or patients, in particular those addressing issues of how to present PRO data. The literature search was carried out in Medline combining search components for PROs and feedback reports in PubMed. For the search component ‘PRO’, we used the Mesh terms ‘Patient Outcome Assessment’, ‘Patient Reported Outcome Measures’ and ‘Quality of Life’ and combined these by use of the Boolean operator ‘OR’. For the search component ‘feedback report’, we used the Mesh terms ‘Electronic Health Records’, Health Smart Cards’ and ‘Patient Portals’ and the entry terms ‘patient feedback report’ and ‘feedback report’ in the title and abstract and combined these by use of the Boolean operator ‘OR’. Both search components were combined by use of the Boolean operator ‘AND’ and filtered by language (German and English) and by studies concerning human subjects. We screened title and abstracts of the identified publications and reviewed full text of those meeting our inclusion criteria. We also screened general scientific literature on how to present data. In addition, members of the special interest group ‘Quality of Life in Clinical Practice’ of the International Society of Quality of Life Research (ISOQOL) were approached regarding any (personal) experience, articles, guidelines etc. on how to feedback PROs to patients.

In a second step, we developed two preliminary versions of a feedback report (a graphical display and a text-based version) relating to the PROMIS® domains of Physical Function and Pain Interference. Our research group included three experts experienced in quality of life and PRO research with an additional clinical background (a psychologist, a physician specialized in internal medicine and psychosomatic medicine and a nurse). As part of the development, we discussed what information to include in the reports based on the results of the literature review and previous experience with PRO feedback reports for health care professionals, followed by initial drafts and two revision rounds.

In a third step, the two preliminary report versions were discussed with patients in terms of the value of the information, comprehension and interpretation of the report using qualitative methods (semi-structured interviews including cognitive debriefing techniques). The methodological approaches are explained in more detail later.

**Fig. 1:**
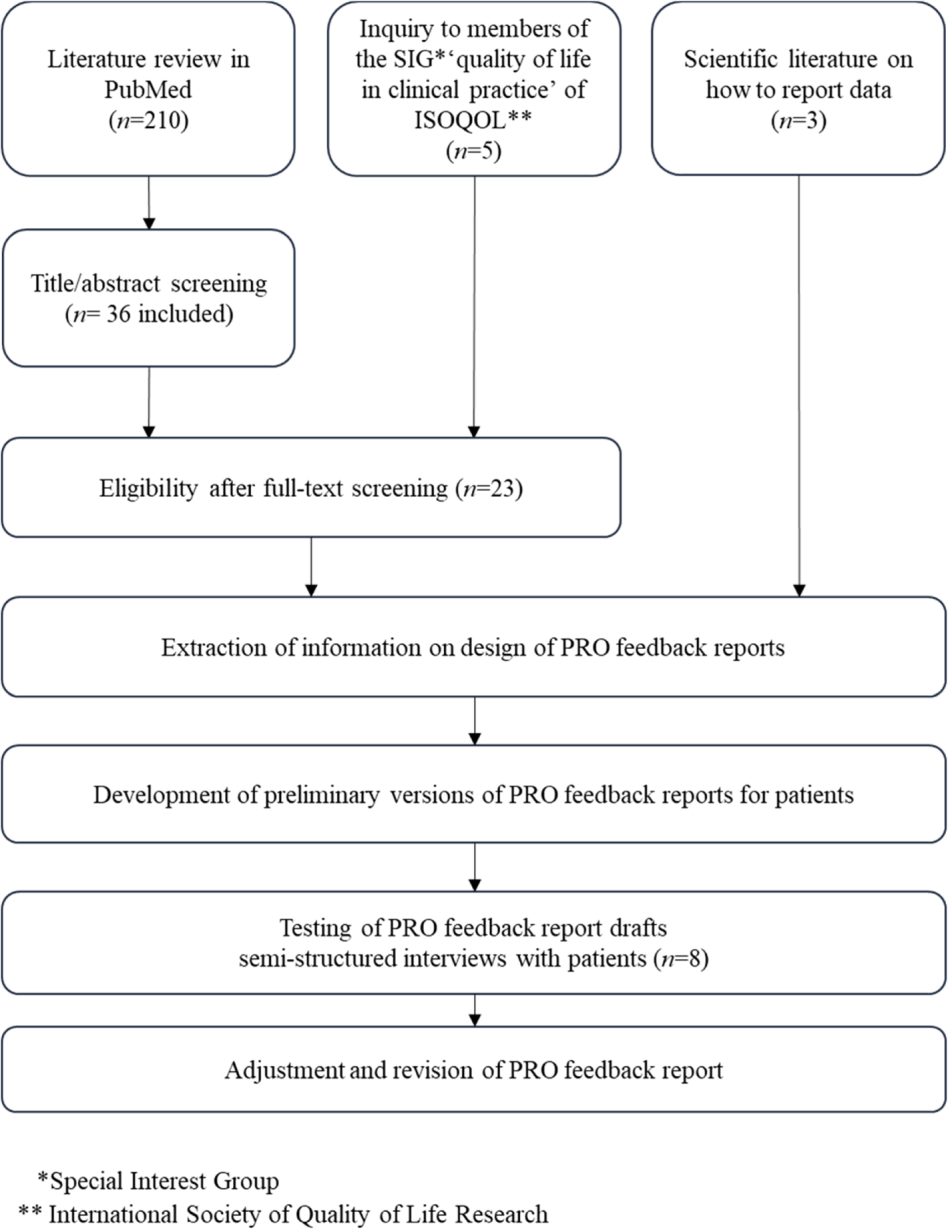
PRO feedback report development process – flow chart

Finally, we derived reasonable modifications for the PRO feedback report based on the results of the interviews and revised the PRO feedback report versions for patients according to the results of the interviews with patients. We discussed these in light of results of the literature search. An overview of the development process is provided in Fig. 1.

### Patient interviews

Orthopedic (primary knee arthroplasty) patients at a university hospital in Germany were invited to participate in our study (convenience sampling) 1 to 2 days after surgery. The sample size was not predetermined, as we were aiming to reach data and thematic saturation [24]. We therefore continued recruitment until no additional codes or categories emerged from the interviews (thematic/data saturation).

Patients were eligible to participate if they were over 18 years, had sufficient knowledge of German (spoken and written), were able to understand the purpose of the study and gave written informed consent. Patients with cognitive limitations, or insufficient knowledge of German were excluded from the study. Ethical approval was obtained from the ethics committee of the university hospital.

The first author conducted all semi-structured interviews with patients according to the interview guideline during the hospital stay. Patients received printed copies of both report versions for both health domains in random order, as the patient app does not currently include any feedback report for patients. They were initially invited to look at the paper copies of the feedback report on their own. Cognitive debriefing methods for probing such as think-aloud and comprehension were used [25]. To assess comprehension of the feedback report drafts, patients were engaged to explain what they see, how they interpret the different elements, and what information they get out of the report. If an element of the report was not addressed by a patient, we explicitly pointed it out. Further, patients were asked which report style they prefer and if a feedback report would be of value and why.

Interviews were tape recorded and transcribed verbatim. All interviews were analyzed using a content analysis approach. Based on the guideline developed for the interviews, we pre-specified a category structure (Table 4) that is ‘general value of the report’, ‘favored report style’ and ‘comprehension’ and ‘informative content’ of both report styles. Additional categories were created, if necessary. All interview transcripts were read multiple times and codes were extracted and assigned to one of the specified categories. We subsequently summarized and discussed the pre-defined and created categories and included codes in detail. The interview analysis was performed using MAXQDA^10^ software for Windows.

## Results

### Development process of a PRO feedback report for patients

Review of the literature revealed only few studies focusing on PRO feedback reports in general, and on how to report PRO scores in particular. Results from previous studies showed that patients prefer short and condensed information [26] using simple language [27]. An efficient way to present longitudinal PRO scores is by use of graphs such as bar charts or line graphs [28–39], alternatively PRO results could be reported by use of tables [40, 41] or textual reports [31, 39]. A text-based report is the least preferred option for patients [31] but is less susceptible to misinterpretation [39]. For graphs, meaningful labelling of axes is recommended. For example, descriptive labeling, and harmonization of directionality, where higher indicates better facilitates interpretation of PRO scores [35–37, 41, 42]. Further, highlighting results of importance helps to focus attention and support the interpretation of results [30, 35, 36]. Color-shading, threshold lines or circles around important scores are suggested to increase readability and interpretability of graphical displays [14, 15, 29, 35, 36]. Additional information such as comparison to a reference population could be of value for some patients, but might be critical to others [34, 41, 43]. An overview of the results of the literature review is provided in Table 1.

**Table 1:** PRO feedback reports - literature overview

First author, year [Ref]	Target group	Main findings regarding PRO feedback reports
Aldekhyyel R.N., 2018 [44]	health care professionals, patients	PRO results are reported to health care professionals, text-based messages to patients
Baldwin J.L., 2017 [27]	health care professionals, patients	results have to be easy to understand, i.e. simplified medical terms, flagging results by including a short explanation / interpretation
Bantug E.T., 2016 [28]	health care professionals, patients	simple graphs (on a domain level) are less prone to misinterpretation, results over time are of value to patients, additional score information, e.g. *CI* or *SE* are less preferred by patients, important findings should be highlighted, text should complement PRO scores, to many details increase cognitive burden
Barthel D., 2016 [14]	health care professionals	colors-coding helps to interpret results, it is important to present results over time
Brundage M., 2005 [39]	patients	text-based reports are the least preferred report style, simple line graphs to present longitudinal data are preferred by the majority of patients, additional information, such as error bars did not improve preference of patients
Cronin R.M., 2018 [31]	patients	simple graphs including text is preferred over text only feedback, textual reports were less susceptible to misinterpretation compared to complex graphs sharing results with health care professionals is important to patients
Demiris G., 2011 [32]	health care professionals, patients	graphs are easier to understand than text-based feedback
Fried T.R., 2016 [26]	patients	patients prefer short and condensed information
Fritz F., 2011 [40]	health care professionals	overview of results in table format
Gilbert A., 2015 [29]	health care professionals	tables or graphs (e.g. bar graphs) to present PROs over time, scores could be shown on an item or domain level, information on clinical importance of scores or clinically important change aid decision-making, cut-off line to indicate significant scores
Grossman L.V., 2017 [43]	health care professionals, patients	depending on the population – reference to the norm population might be beneficial to patients
Harle C.A., 2016 [45]	health care professionals	PRO scores are presented on 0–100 scale including nominal results such as mild or severe
Izard J., 2014 [33]	health care professionals, patients	information that should be provided in a feedback report: comparison to the patient population, individual before and after treatment comparison, prediction of future scores, graphical display (bar or line chart) or tables are favored over pictographs, patients prefer bar charts, health care professionals prefer tables, bar charts and line graphs equally, reference to comparison groups could be concerning, dynamic display of reports to be able include or exclude additional elements might be useful
Krogstad H., 2017 [46]	health care professionals	display of PRO scores either in fixed order or showing the most alarming on top
Krogstad H., 2019 [42]	health care professionals	scales with anchored text facilitates interpretation of results
McNair A.G., 2010 [34]	patients	simple line graphs on domain level are easy to understand
Rothrock N.E., 2019 [15]	health care professionals	scores are shown in a longitudinal graph, results on an item level are provided for the most recent assessment
Schwartzberg L., 2016 [47]	health care professionals	graphical and numerical presentation of longitudinal PRO scores
Smith K.C., 2016 [35]	health care professionals, patients	patients and health care professionals prefer line graphs (patients prefer simple graphs (including simplified language), health care professionals prefer more detail including *CI*, *p*-values etc.), confusion if higher scores indicate better or worse outcomes should be avoided - y-axis should include descriptive labels and / or numbers, possible concerning scores and change should be highlighted (shading the normal range green, shading concerning scores red, red circles or threshold lines)
Snyder C.F., 2019 [37]	health care professionals, patients	PRO scores should be presented on domain level over time using line graphs, clear labeling of axis (descriptive labeling) facilitates interpretation, concerning results should be highlighted
Snyder C.F., 2017 [36]	health care professionals, patients	directionality of graphs with higher scores meaning better outcome are less prone to misinterpretation, threshold lines or red circles seem to be easier to understand compared to a green shaded area indicating the normal range
Sokka T., 2016 [48]	health care professionals	patients’ responses are shown on an item level
Wu A.W., 2016 [41]	health care professionals	health care professionals prefer tables or graphs to display longitudinal results, clarification if higher scores mean better or worse outcomes by adding meaning of scores to graphs and arrows indicating the direction of scores, information on normal range, meaning of scores and guidance for action would be helpful, patients appreciate viewing own results and want to be notified when health care professionals view the scores

In the initial development phase, we decided to provide feedback reports on a domain level (PROMIS® health domains Physical Function and Pain Interference), rather than a combined report, to increase readability on mobile devices, which was in concordance with the results of the literature review. We were aiming to include the feedback reports in an existing patient app that already includes a message function. Thus, we decided to develop two different report styles, a text-based report, which could be sent like using the message function, and a graphical display, which would be shown after the PROM completion. The text-based report was considered a pragmatic solution and could be implemented without additional effort, whereas, reprogramming would be needed to incorporate the graphical display. Our research team determined the following information to include in the PRO feedback report: the domain T-score, reference to the norm population, score thresholds and the predicted disease trajectory, as well as the opportunity to share information with health care professionals by use of the text message function or by a push button.

The text-based version included general information regarding the assessment, estimation of the current individual level of symptoms or function (including examples of limitations), and reference to the norm population. Patients were informed to contact their health care team in case of unexpected PRO changes or related questions. We used the thresholds provided by the PROMIS® Health Organization [49] to categorize value ranges. We created four categories for the Pain Interference health domain (group 1: T-score < 55; group 2: T-score of 55–60; group 3: T-score of 61–70; group 4: T-score > 70) and five categories for Physical Function (group 1: T-score < =30; group 2: T-score of 31–40; group 3: T-score of 41–45; group 4: T-score of 45–65; group 5: T-score > 65). Text messages were adapted according to the respective category. Patients scoring within the range of a category would receive the corresponding text message.

**Fig. 2:**
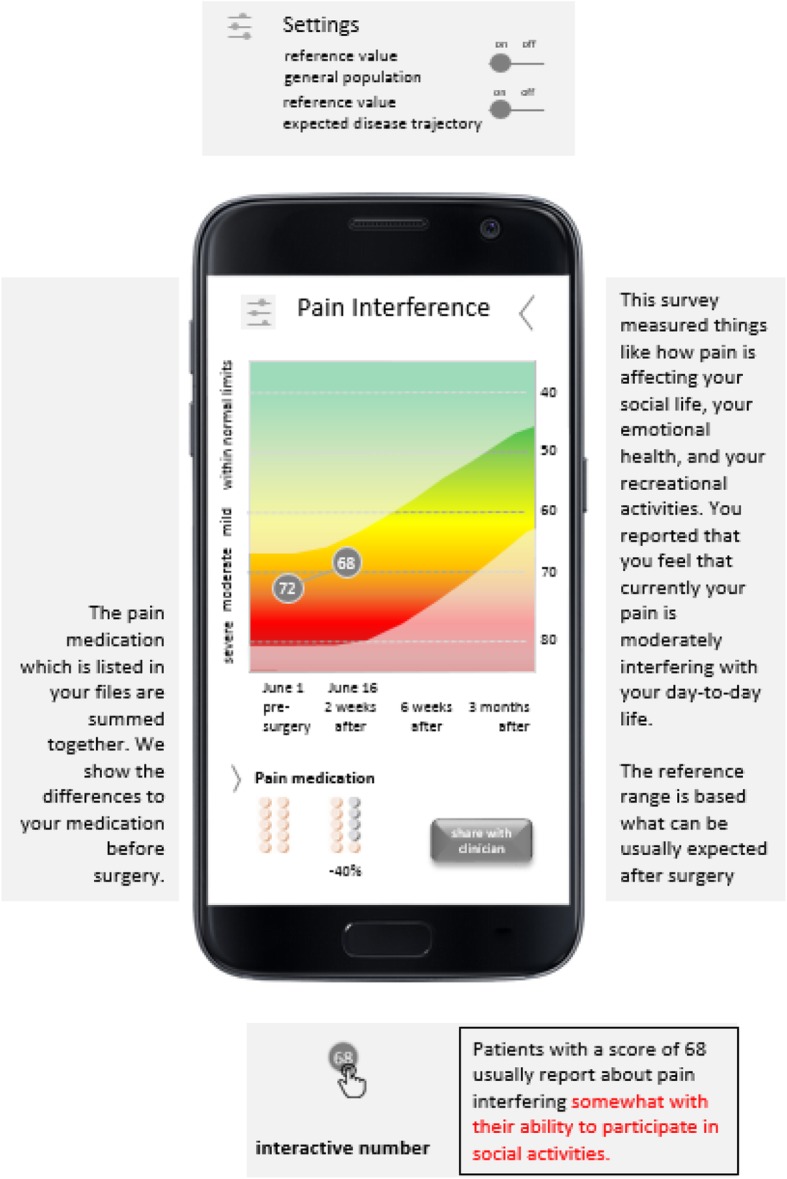
Preliminary version of the graphical PRO feedback reports for the health domain of Pain Interference

The graphical display included information on the individual T-score, the reference to the norm population, score thresholds and information on the predicted disease trajectory. The information was included on a line graph, where scores are plotted over time. On the x-axis, the measurement date as well as the time passed since the knee arthroplasty are displayed. We included two y-axis. The right-hand side y-axis only included the numerical scale. Descriptive labels were added to the left-hand side y-axis, referring to norm population scores. In addition, we added a rainbow-colored background from red (bottom) to green (top) to the line chart to visualize the grading of the individual scores. Whereas higher scores of Physical Function are better outcomes, higher scores of Pain Interference are worse outcomes. In order to account for this, we reversed the scale on the right y-axis for Pain Interference to harmonize the direction of the line graphs, i.e. the higher the better. We overlaid the line graph with an additional shaded area indicating the projected trajectory of the health domain. A button to share the information with health care professionals was included. For Pain Interference, we added information on the relative frequency of pain medication according to measurement points and percentage of change in pain medication in reference to the previous value. For Physical Function, we added information on immune activity including a rating of inflammatory markers (low, medium or high). The preliminary versions of the PRO feedback reports for the domain Pain Interference are displayed in Fig. 2 and Table 2.

**Table 2:** Preliminary version of the text-based PRO feedback report for the health domain of Pain Interference according to grouped T-score categories

Group 1: T-score < 55	“You took a survey this week called “Check-In: Your Pain” that measured things like how pain is affecting your social life, your emotional health, and your recreational activities. You indicated that pain is not or is hardly interfering with your day-to-day life. Your total score is similar to the average score of the population. If you have questions about this survey, please reach out to your care team.”
Group 2: T-score of 55–60	“You took a survey called “Check-In: Your Pain” that measured things like how pain is affecting your social life, your emotional health, and your recreational activities. You reported that you feel that pain is slightly interfering with your day-to-day life. Around one third of the population report a similar level of pain. If you have questions about this survey, please reach out to your care team.”
Group 3: T-score of 61–70	“You took a survey called “Check-In: Your Pain” that measured things like how pain is affecting your social life, your emotional health, and your recreational activities. Your reported that you feel that pain is considerably interfering with your day-to-day life. Around 20% of the population report a similar level of pain. This may be due to your recent knee replacement or other health issues. If you have questions about this survey, please reach out to your care team.”
Group 4: T-score > 70	“You took a survey called “Check-In: Your Pain” that measured things like how pain is affecting your social life, your emotional health, and your recreational activities. Your score means that you feel that pain is severely interfering with your day-to-day life. Less than 5% of the population report a similar level of pain. This may be due to your recent knee replacement or other health issues. If you have questions about this survey, please reach out to your care team.”

### Patient interviews

We invited 15 adult patients, who have underwent knee arthroplasty to participate in our study between September 2018 and January 2019. Seven patients who declined to participate said they were not interested in the study or were already participating in another research study. Non-responders were predominantly male (*n* = 6). We interviewed eight patients after knee arthroplasty, five women (62.5%) and three men (37.5%) aged between 60 and 80. We continued interviews until generated data was repetitive and no new codes or categories emerged during the analyses. This was the case after five interviews. The interviews took 24 min and 33 s on average, ranging from 19:59 to 28:38 min. Sociodemographic details of the study participants are provided in Table 3.

**Table 3:** Sociodemographic details (*n* = 8)

Age (mean)	72.25
Sex (female, %, (n))	62.5% (5)
Relationship Status (%, (n))	
single	0% (0)
married/in a relationship	87.5% (7)
separated/divorced	0% (0)
widowed	12.5% (1)
Level of Education	
less than secondary education	0% (0)
lower secondary education	75% (6)
upper secondary education	12.5% (1)
tertiary education or higher	12.5% (1)
Working Status	
working	12.5% (1)
retired	87.5% (7)
Household Income (before tax)	
€0 – €19,999	0% (0)
€20,000 - €39,999	37.5% (3)
€40,000 - €59,999	25% (2)
€60,000 - €79,999	0% (0)
prefer not to say	37.5% (3)

To achieve the aim of the study and the purpose of the interviews, we predefined four categories and four sub-categories, which were *general value of the report*, *favored feedback report style*, *graphical display*, including the sub-categories *comprehension* and *informative content*, and *text-based report* including the sub-categories *comprehension* and *informative content*. During the coding process, the additional category *aspects related to the individual person or situation* emerged.

The *general value of the reports* was rated as moderate. While all patients thought such a report would be interesting and they would look at it, only one patient saw personal gain from using the report as a communication tool with health care professionals and as a self-monitoring tool. One participant stated that the majority of people would not understand what this report is about. Another participant stated that the idea of such a feedback report is good, but probably more suited to a younger generation. Another aspect mentioned by one participant was the course of treatment after knee surgery in Germany. This includes an extensive rehabilitation phase in a specialized clinic with daily treatments and regular examinations. The participant questioned the benefit of such a report given the continuous monitoring of the recovery process in Germany.

Participants had different opinions regarding the *favored feedback report style*. Three participants were in favor of the graphical display and two participants were undecided but tended toward the text-based version. Participants in favor of the graphical display argued that it is easy and quick to get the relevant information from the line graph. Those undecided stated that the graphical display looks more appealing, but the text-based version is easier to understand and most people are used to read short text messages.

The *text-based report* was easy to understand and none of the patients had questions regarding comprehension. Participants assessed the report as informative, but thought that some information was repetitive. Issues regarding the reference to the norm population were raised, as the percentage of people reporting similar symptoms or functional limitations would not be relevant. Difficulties in assigning a meaning to the number was also raised. One patient was even put off by it, feeling it would put certain pressure on someone to belong to the majority. Overall, the text-based version was considered to be too long, Participants therefore suggested shortening the text.

The *graphical display* of the reports was perceived to be complex due to the inclusion of a lot of information. Participants were concerned that this format may not be appropriate for an older generation. In terms of comprehension, all participants correctly understood the line graph and were able to interpret the scores. However, some patients needed some initial guidance on how to read a line graph. The rainbow-colored background was understood by all participants. With one exception, participants automatically attached value to the different shades of color. However, the shaded area indicating the projected trajectory of the health domains was not clear to patients. One participant did not ascribe any value to this area. Others understood this area as the range of possible scores, or as a way to draw attention to the area of the respective scores. Just two respondents interpreted the improvement over time as a prognosis for recovery after surgery. Only one participant understood the additional information regarding pain medication and related the information to the Pain Interference T-score. The additional information on immune activity was deemed to be too technical. Participants guessed that it might be related to the immune system, but could not explain what it meant, or relate this information to the Physical Function health domain. The function to share the information with health care professionals was clear to all patients.

During the interviews, five participants emphasized the relevance of *aspects related to the individual person or situation*. It was thought that a comparison to the norm population would not be of particular interest to patients after knee arthroplasty, as the individual before and after comparison of physical function or level of pain is of particular importance. Furthermore, reference to the knee replacement would help to relate the report to the individual patient’s situation. Statements like the activities which one might not be able to do due to the limitations of physical function might not reflect the individual’s activities. The report therefore loses the personal touch. One patient mentioned that the measurement time points should be flexible so that the patient can decide on the frequency of assessments.

An overview of the categories including the number of codes per category and examples of codes is presented in Table 4.

**Table 4:** Coding structure of the interviews, number of codes and coding example

Categories and Sub-categories	Number of codes	Selected codings^a^
General value of the report	8	“If I would have had such a thing, where I could enter my level of pain and my physical activity, I would have had surgery 3 months earlier instead of limping around and causing more damage.” “The idea is good, but maybe more suitable for patients, which are 30 years younger – they might understand it better.” “I would not need it, meaning that I would not invest a lot of time or thought into it.”
Favored feedback report style	5	“The graphical display is just one look and you get it.” “The text-based version was simpler.” “I think the graphical display is better, but this might be related to age.”
Individual-related aspects	8	“I am not interested in it (comparison to norm population). It might be of interest for research, but for me as a patient – no.” “Some text messages relate to knee replacement, I feel addressed by this. It would be important to me that it relates to the knee.”
Graphical display		
Comprehension	64	“For that, I need a little longer …” “Given these points, I would say this person has severe to moderate pain.” “Ah okay, if you once understood the concept ….” “Yes, so red means “danger” or negative and green is obviously the normal range that is understandable. In the middle, it is yellow. Yes, it is comprehensible for me.”
Informative content	16	“It is a lot of information.” “I have to say that I am overwhelmed by it.” “I ask myself, if the time between June 1 and June 16, is not it too long, just based on experience. I have no opinion on it, if one should ask more often or not, I am not sure about it. It would be good if the patient could decide on his own.”
Text-based display		
Comprehension	8	“It is okay and comprehensible.” “No, I have no questions regarding comprehension or things that are unclear.”
Informative content	18	“Parts of the text are repetitive, and could be deleted. The part with the norm population – I do not need such information, I am good with my pre - post comparison.” “The reference might be relevant for the physician, but not for me. If I am in pain, I am in pain and how I deal with it is something different, if 20% or 19% of people have the same level of pain is not relevant for me.” “It is a lot to read, there are a lot of information.”

^a^All interviews were conducted in German and the content translated into English

### Revisions of PRO feedback reports for patients

As a result of the interview analysis, we modified the PRO feedback reports for patients to increase comprehension and the value of the reports. Minimal adjustments were done to the text-based version. This version was comprehensible to all patients, but the text was too long and some of the information was not seen as valuable to patients. We deleted the reference to the norm population. A sentence stating what the score measured as well as a sentence linking the assessment to the patient’s recent knee arthroplasty were added.

**Fig. 3:**
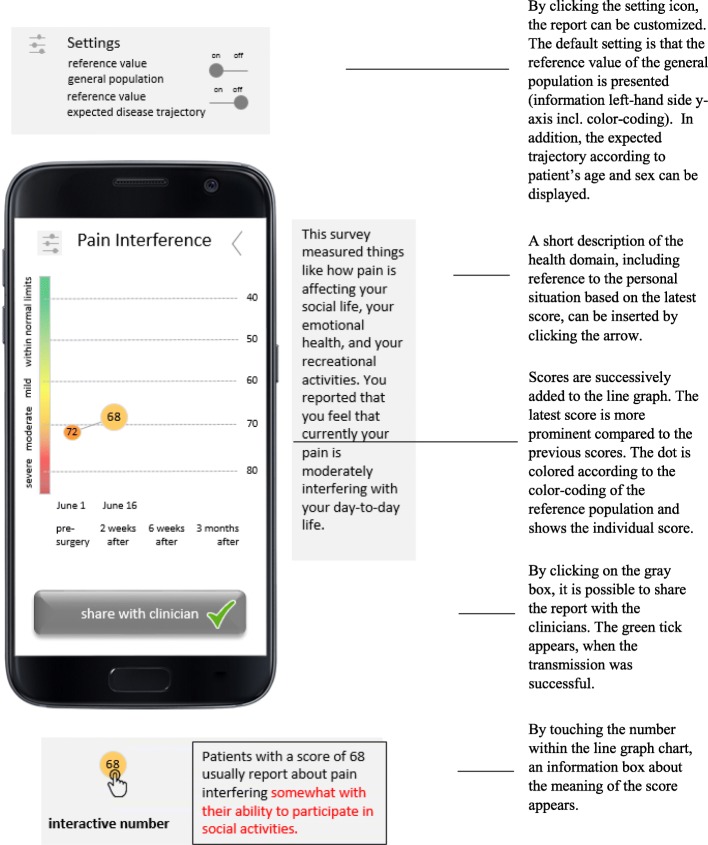
Final graphical display of the PRO feedback reports for the health domain of Pain Interference

The graphical display required more extensive revisions to decrease complexity and thereby increase comprehension and value. We excluded the additional information regarding immune activity and pain medication as this information was misunderstood or not understood by patients and was not interpreted in reference to the measured health domain. The button to share the information with clinicians was enlarged to improve user-friendliness. In addition, we added a green tick to confirm that data were transmitted successfully. We reduced clutter within the line graph to increase readability and comprehension. The reference curve of the expected disease trajectory became an optional setting. The rainbow-colored background was reduced to a bar on the left-hand side y-axis. The data points plotted on the line graph include the scores, as well as the color corresponding to the rainbow-colored bar added to the y-axis. In addition, we decided to enlarge the most recent score to draw attention to it. The revised graphical display for the Pain Interference domain is depicted in Fig. 3.

## Discussion

This study aimed to develop a PRO feedback report for adult knee arthroplasty patients that is easy to understand while providing valuable information. Results showed that the group of knee arthroplasty patients is heterogeneous in terms of age and experience with mobile devices and so are their demands on a PRO feedback report.

Based on the patient interviews, it was not possible to appoint a preferred report style. In previous studies, patients preferred graphical displays over a text-based report [31, 39]. Patients reported that the graphical display looked more appealing but the text-based feedback report version was easy to understand, even if partly too lengthy. Corresponding with our finding, Fried and colleagues [26] reported that feedback reports for patients should contain short and useful information instead of lengthy explanations.

Presenting data over time allows users to evaluate their individual progress and to compare current and previous scores. Patients valued the line graph presenting scores over time as important information, something which is not provided in our text-based version. This finding corresponds to the results reported by Brundage et al. [30] that patients preferred the depiction of multiple time points.

Interestingly, participants considered the reference to the norm population in the text-based version to not be of value and hence not relevant. However, they viewed the labels added to the y-axis and the color-coding within the graphical display positively. This implicit reference to the norm population supports the evaluation of individual domain scores. Similar findings were reported in previous studies that the inclusion of information referring to a reference group, such as labels of axis or integration of thresholds or a norm line, is helpful in evaluating the results [15, 29, 33, 36].

During the interviews, initial guidance on how to read the line graph was helpful to patients, and they suggested that some guidance should be given to all users before receiving the feedback report. Baldwin and colleagues [27] outlined that information should be provided in a simple way, including a simple interface design. This means that if additional guidance is required, the interface design might be too complex. However, a brief introduction to the PRO feedback report, including the functionality of the graphical display, could be implemented in the mobile app. The user could refer back to this information when needed. This training on how to use and interpret the feedback report could help to increase the utilization of the report among users [50].

The graphical display of the feedback report included additional information that overwhelmed participants. Considering the conceptual framework ‘mHealth for older users’ described by Wildenbos et al. [51], vision problems, cognitive restriction as well as attitudes and health-technology literacy might have influenced the perceived overload of the graphical display. Further, the use of medical terms instead of layman’s terms and the presentation of the clinical information (pain medication and immune activity) was difficult for participants to understand and prone to misinterpretation. This information was removed from the report to ensure that appropriate information is provided according to the health literacy level of patients using simple language, crucial aspects for a user-friendly interface [27, 51].

Another aspect study participants addressed during the interviews was the penetration of smartphone use within this specific target group. Only a few study participants had a smartphone and only one patient stated that he uses his smartphone for purposes beyond communication (phone calls and messages). When designing PRO feedback for mobile app use, two major challenges have to be considered. Firstly, the penetration of smartphone use in a specific age group has to be evaluated in a national context [52, 53]. Secondly, experience and user behavior with mobile devices is closely linked to the health-technology literacy level of the target group [51, 54, 55]. The field of mobile applications offers technological opportunities such as customizations and user guidance. In this way, it is possible to adjust the default settings of the graphical design of the report so that the interested and experienced user can fade-in additional information, but the default design does not overwhelm the inexperienced user [33].

### Limitations

This study has some limitations that have to be considered with regard to the study results. There were some limitations to the study design. We did not include patient research partners in the study and discussed only the two developed feedback report styles with patients without testing alternative design formats including less or more information. The information included in the feedback report and the design would have been different by including patient research partners within the study. Furthermore, there would have been different formats to present the information, especially for the graphical display of the report. Thus, we are not able to make any statement in terms of the optimal design of PRO feedback reports for patients. However, the study’s goal was to develop a feedback report for mobile devices that provides valuable information to patients after knee arthroplasty, while being easy to understand and to evaluate. We have to test the developed feedback report to evaluate functioning and the practical value of sharing such PRO information with patients in future studies. A feasibility study to investigate how frequently patients look at the report and how the information facilitate communication between patients and health care professionals during the recovery period is needed.

We initially planned to conduct focus groups instead of semi-structured interviews to induce discussions among participants. We invited patients to take part in a focus group, but all patients preferred an individual interview. The coding was conducted by one researcher only. To increase reliability, all codes were discussed with a second researcher and if necessary, codes were removed from a category or assigned to another.

In addition, we were faced with a high non-responder rate in our study and recruited a small number of participants. However, we consider the number of patients to be acceptable given the narrow scope of the study and the saturation of data after five interviews. Data generated in the last interviews were repetitive and no new codes or categories emerged, which lead to the assumption of data and thematic saturation [24]. Nevertheless, the results of this study have to be interpreted cautiously. The findings are limited to the specific setting of the study and cannot be generalized.

## Conclusion

The development of a PRO feedback report for knee arthroplasty patients for mobile app use is challenging due to the heterogeneous group of patients. The range of health-technology literacy levels within the target group has to be considered when developing an easy to understand and informative PRO feedback report for patients. The amount of information provided to patients has to be balanced to minimize the complexity of the report while maximizing the value for patients. A graphical display including short explanatory texts seems to be the most promising approach as a simple default version can be displayed, while additional information can be set as supplementary information for interested patients.

## Data Availability

The dataset used and analyzed during the current study is available from the corresponding author on reasonable request.

## Abbreviations

PRO: Patient-reported outcomes
PROM: Patient-reported outcomes measure
PROMIS®: Patient-Reported Outcomes Measurement Information System

## References

[1] Higginson IJ, Carr AJ (2001). Measuring quality of life - using quality of life measures in the clinical setting. Br Med J.

[2] Greenhalgh J (2009). The applications of PROs in clinical practice: What are they, do they work, and why?. Quality of life research : an international journal of quality of life aspects of treatment, care and rehabilitation.

[3] Greenhalgh, J., Dalkin, S., Gooding, K., Gibbons, E., Wright, J., Meads, D., Black, N., Valderas, J. M., & Pawson, R. (2017). Health services and delivery research. In: Functionality and feedback: A realist synthesis of the collation, interpretation and utilisation of patient-reported outcome measures data to improve patient care. *NIHR Journals Library* Copyright (c) Queen's Printer and Controller of HMSO 2017. This work was produced by Greenhalgh et al. under the terms of a commissioning contract issued by the Secretary of State for Health. This issue may be freely reproduced for the purposes of private research and study and extracts (or indeed, the full report) may be included in professional journals provided that suitable acknowledgement is made and the reproduction is not associated with any form of advertising. Applications for commercial reproduction should be addressed to: NIHR Journals Library, National Institute for Health Research, Evaluation, Trials and Studies Coordinating Centre, Alpha House, University of Southampton Science Park, Southampton SO16 7NS, UK., Southampton (UK). 10.3310/hsdr05020. 28121094

[4] Snyder CF, Jensen RE, Segal JB, Wu AW (2013). Patient-reported outcomes (PROs): Putting the patient perspective in patient-centered outcomes research. Med Care.

[5] Rose M, Bezjak A (2009). Logistics of collecting patient-reported outcomes (PROs) in clinical practice: An overview and practical examples. Quality of life research : an international journal of quality of life aspects of treatment, care and rehabilitation.

[6] Haverman L, van Rossum MAJ, van Veenendaal M, van den Berg JM, Dolman KM, Swart J, Kuijpers TW, Grootenhuis MA (2013). Effectiveness of a web-based application to monitor health-related quality of life. Pediatrics.

[7] Malvey D, Solovnsky D (2014). mHealth: Transforming healthcare.

[8] Weinstein RS, Lopez AM, Joseph BA, Erps KA, Holcomb M, Barker GP, Krupinski EA (2014). Telemedicine, Telehealth, and Mobile health applications that work: Opportunities and barriers. Am J Med.

[9] Dobkin BH, Dorsch A (2011). The promise of mHealth: Daily activity monitoring and outcome assessments by wearable sensors. Neurorehabil Neural Repair.

[10] Hamine S, Gerth-Guyette E, Faulx D, Green BB, Ginsburg AS (2015). Impact of mHealth chronic disease management on treatment adherence and patient outcomes: A systematic review. J Med Internet Res.

[11] Jensen RE, Gummerson SP, Chung AE (2016). Overview of patient-facing Systems in Patient-Reported Outcomes Collection: Focus and Design in Cancer Care. Journal of oncology practice.

[12] Jiang J (2018). Millenials stand out for their technology use, but older generations also embrace digital life.

[13] Joe J, Demiris G (2013). Older adults and mobile phones for health: A review. J Biomed Inform.

[14] Barthel D, Fischer KI, Nolte S, Otto C, Meyrose AK, Reisinger S, Dabs M, Thyen U, Klein M, Muehlan H, Ankermann T, Walter O, Rose M, Ravens-Sieberer U (2016). Implementation of the Kids-CAT in clinical settings: a newly developed computer-adaptive test to facilitate the assessment of patient-reported outcomes of children and adolescents in clinical practice in Germany. Quality of life research : an international journal of quality of life aspects of treatment, care and rehabilitation.

[15] Rothrock NE, Bass M, Blumenthal A, Gershon RC, Hanson B, Joeris A, Kaat A, Morrison S, O'Toole RV, Patel S, Stover M, Weaver MJ, White R, Varela Diaz M, Vrahas MS (2019). AO patient outcomes center: Design, implementation, and evaluation of a software application for the collection of patient-reported outcome measures in orthopedic outpatient clinics. JMIR formative research.

[16] Auyong DB, Allen CJ, Pahang JA, Clabeaux JJ, MacDonald KM, Hanson NA (2015). Reduced length of hospitalization in primary Total knee Arthroplasty patients using an updated enhanced recovery after orthopedic surgery (ERAS) pathway. J Arthroplast.

[17] Kumar PJ, McPherson EJ, Dorr LD, Wan Z, Baldwin K (1996). Rehabilitation after total knee arthroplasty: A comparison of 2 rehabilitation techniques. Clin Orthop Relat Res.

[18] Chapter 3: A practical guide to improving patient outcomes (2000). Orthopedic nursing 19 Suppl:22–28. 11153498

[19] Holman H, Lorig K (2004). Patient self-management: A key to effectiveness and efficiency in Care of Chronic Disease. Public Health Rep.

[20] Cameron A (1998). Patient self-management. PharmacoEconomics & Outcomes News Weekly.

[21] Bodenheimer T, Lorig K, Holman H, Grumbach K (2002). Patient self-management of chronic disease in primary care. JAMA.

[22] Kim K, Pham D, Schwarzkopf R (2016). Mobile application use in monitoring patient adherence to perioperative Total knee Arthroplasty protocols. Surgical technology international.

[23] Santana MJ, Feeny D (2014). Framework to assess the effects of using patient-reported outcome measures in chronic care management. Quality of life research : an international journal of quality of life aspects of treatment, care and rehabilitation.

[24] Saunders B, Sim J, Kingstone T, Baker S, Waterfield J, Bartlam B, Burroughs H, Jinks C (2018). Saturation in qualitative research: Exploring its conceptualization and operationalization. Qual Quant.

[25] Collins D (2003). Pretesting survey instruments: An overview of cognitive methods. Quality of life research : an international journal of quality of life aspects of treatment, care and rehabilitation.

[26] Fried TR, Redding CA, Robbins ML, Paiva AL, O'Leary JR, Iannone L (2016). Development of personalized health messages to promote engagement in advance care planning. J Am Geriatr Soc.

[27] Baldwin JL, Singh H, Sittig DF, Giardina TD (2017). Patient portals and health apps: Pitfalls, promises, and what one might learn from the other. Healthcare (Amsterdam, Netherlands).

[28] Bantug ET, Coles T, Smith KC, Snyder CF, Rouette J, Brundage MD (2016). Graphical displays of patient-reported outcomes (PRO) for use in clinical practice: What makes a pro picture worth a thousand words?. Patient Educ Couns.

[29] Gilbert A, Sebag-Montefiore D, Davidson S, Velikova G (2015). Use of patient-reported outcomes to measure symptoms and health related quality of life in the clinic. Gynecol Oncol.

[30] Brundage MD, Smith KC, Little EA, Bantug ET, Snyder CF (2015). Communicating patient-reported outcome scores using graphic formats: Results from a mixed-methods evaluation. Quality of life research : an international journal of quality of life aspects of treatment, care and rehabilitation.

[31] Cronin RM, Conway D, Condon D, Jerome RN, Byrne DW, Harris PA (2018). Patient and healthcare provider views on a patient-reported outcomes portal. Journal of the American Medical Informatics Association : JAMIA.

[32] Demiris G, Thompson H (2011). Smart homes and ambient assisted living applications: From data to knowledge-empowering or overwhelming older adults? Contribution of the IMIA smart homes and Ambiant assisted living working group. Yearbook of medical informatics.

[33] Izard J, Hartzler A, Avery DI, Shih C, Dalkin BL, Gore JL (2014). User-centered design of quality of life reports for clinical care of patients with prostate cancer. Surgery.

[34] McNair AG, Brookes ST, Davis CR, Argyropoulos M, Blazeby JM (2010). Communicating the results of randomized clinical trials: Do patients understand multidimensional patient-reported outcomes?. Journal of clinical oncology : official journal of the American Society of Clinical Oncology.

[35] Smith KC, Brundage MD, Tolbert E, Little EA, Bantug ET, Snyder CF (2016). Engaging stakeholders to improve presentation of patient-reported outcomes data in clinical practice. Supportive care in cancer : official journal of the Multinational Association of Supportive Care in Cancer.

[36] Snyder CF, Smith KC, Bantug ET, Tolbert EE, Blackford AL, Brundage MD (2017). What do these scores mean? Presenting patient-reported outcomes data to patients and clinicians to improve interpretability. Cancer.

[37] Snyder C, Smith K, Holzner B, Rivera YM, Bantug E, Brundage M (2019). Making a picture worth a thousand numbers: Recommendations for graphically displaying patient-reported outcomes data. Quality of life research : an international journal of quality of life aspects of treatment, care and rehabilitation.

[38] Wu AW, Kharrazi H, Boulware LE, Snyder CF (2013). Measure once, cut twice--adding patient-reported outcome measures to the electronic health record for comparative effectiveness research. J Clin Epidemiol.

[39] Brundage M, Feldman-Stewart D, Leis A, Bezjak A, Degner L, Velji K, Zetes-Zanatta L, Tu D, Ritvo P, Pater J (2005). Communicating quality of life information to cancer patients: A study of six presentation formats. Journal of clinical oncology : official journal of the American Society of Clinical Oncology.

[40] Fritz F, Stander S, Breil B, Riek M, Dugas M (2011). CIS-based registration of quality of life in a single source approach. BMC medical informatics and decision making.

[41] Wu AW, White SM, Blackford AL, Wolff AC, Carducci MA, Herman JM, Snyder CF (2016). Improving an electronic system for measuring PROs in routine oncology practice. Journal of cancer survivorship : research and practice.

[42] Krogstad H, Sundt-Hansen SM, Hjermstad MJ, Hagensen LA, Kaasa S, Loge JH, Raj SX, Steinsbekk A, Sand K (2019). Usability testing of EirV3-a computer-based tool for patient-reported outcome measures in cancer. Supportive care in cancer : official journal of the Multinational Association of Supportive Care in Cancer.

[43] Grossman LV, Mitchell EG (2017). Visualizing the patient-reported outcomes measurement information system (PROMIS) measures for clinicians and patients. AMIA Annual Symposium proceedings AMIA Symposium.

[44] Aldekhyyel RN, Melton GB, Lindgren B, Wang Y, Pitt MB (2018). Linking pediatrics patients and nurses with the pharmacy and electronic health record system through the inpatient television: A novel interactive pain-management tool. Hospital pediatrics.

[45] Harle CA, Listhaus A, Covarrubias CM, Schmidt SO, Mackey S, Carek PJ, Fillingim RB, Hurley RW (2016). Overcoming barriers to implementing patient-reported outcomes in an electronic health record: A case report. Journal of the American Medical Informatics Association : JAMIA.

[46] Krogstad H, Brunelli C, Sand K, Andersen E, Garresori H, Halvorsen T, Haukland EC, Jordal F, Kaasa S, Loge JH, Lohre ET, Raj SX, Hjermstad MJ (2017). Development of EirV3: A computer-based tool for patient-reported outcome measures in Cancer. JCO clinical cancer informatics.

[47] Schwartzberg L (2016) Electronic patient-reported outcomes: The time is ripe for integration into patient care and clinical research. American Society of Clinical Oncology educational book American Society of Clinical Oncology Annual Meeting 35:e89-e96. doi:10.14694/edbk_158749 10.1200/edbk_158749. 10.1200/EDBK_158749 27249775

[48] Sokka T (2016). Go, go, GoTreatIT!. Clin Exp Rheumatol.

[49] PROMIS Health Organisation (2019). PROMIS Score Cut Points - General guidelines for interpreting PROMIS scores Available via Health Measures.

[50] Amante DJ, Hogan TP, Pagoto SL, English TM (2014). A systematic review of electronic portal usage among patients with diabetes. Diabetes Technol Ther.

[51] Wildenbos GA, Peute LW, Jaspers MW (2015). A framework for evaluating mHealth tools for older patients on usability. Studies in health technology and informatics.

[52] Berenguer A, Goncalves J, Hosio S, Ferreira D, Anagnostopoulos T, Kostakos V (2017). Are smartphones ubiquitous?: An in-depth survey of smartphone adoption by seniors. IEEE Consumer Electronics Magazine.

[53] Sharareh B, Schwarzkopf R (2014). Effectiveness of telemedical applications in postoperative follow-up after total joint arthroplasty. The Journal of arthroplasty.

[54] Grindrod KA, Li M, Gates A (2014). Evaluating user perceptions of mobile medication management applications with older adults: A usability study. JMIR mHealth and uHealth.

[55] Arnhold M, Quade M, Kirch W (2014). Mobile applications for diabetics: A systematic review and expert-based usability evaluation considering the special requirements of diabetes patients age 50 years or older. J Med Internet Res.

